# Sorcery and witchcraft beliefs on the front line of public health response in Papua New Guinea and beyond

**DOI:** 10.5365/wpsar.2024.15.3.1171

**Published:** 2024-09-04

**Authors:** Miranda Forsyth, Joanne Taylor, Tambri Housen, Celeste Marsh, Philip Gibbs, William Kipongi

**Affiliations:** a Australian National University, Canberra, Australia.; b University of Newcastle, Newcastle, New South Wales, Australia.; c Divine Word University, Madang, Papua New Guinea.; d National Research Institute, Port Moresby, Papua New Guinea.

## Abstract

**Problem:**

Many communities refer to sorcery or witchcraft to explain misfortunes such as sickness, death and disability. The effects of these beliefs on public health service delivery have long been overlooked. Beliefs in sorcery and witchcraft are significant challenges for health-care workers to understand to deliver better health outcomes and avoid inadvertently triggering accusations of witchcraft that may lead to violence.

**Context:**

This paper examines the impacts of accusations of sorcery and related violence on the provision of health care in Papua New Guinea.

**Action:**

The discussion focuses on a workshop held in Papua New Guinea in September 2022 with health extension officers on the topic of health-care delivery and sorcery accusations.

**Lessons learned:**

The workshop confirmed the challenges that beliefs in sorcery and witchcraft present for health extension officers and suggested several strategies that could be used to navigate them. It identified several possible future measures that those on the front line of community health-care delivery considered most important in responding to the issue. These included educating health-care workers on how to effectively address sorcery beliefs when delivering health care and developing communication techniques on the causes of death and sickness that avoid triggering sorcery accusations.

**Discussion:**

This paper reviews the findings of the workshop in the broader context of the effects of beliefs in witchcraft on public health delivery globally. Because of the close connections between sorcery beliefs and health, equipping health-care workers and field epidemiologists with strategies to address these beliefs effectively is critical to delivering better health care, facilitating timely response to public health events, and helping to prevent violence related to sorcery accusations. This need exists in all countries where sorcery beliefs related to health, illness, disability and death are prevalent.

## PROBLEM

Across the globe today, many communities refer to sorcery or witchcraft to explain misfortune of all kinds, including sickness, death and disability. (*1*) This is certainly the case in Papua New Guinea, a highly diverse country of approximately 9 million people, where belief in sorcery is widespread across all sectors of society, including among those in leadership positions, the justice sector and health care. (*2*) We discuss the implications of these beliefs for public health professionals. While beliefs in sorcery and witchcraft also play a role in traditional healing practices, our focus is on the harmful results of these beliefs and is not intended to advocate against traditional healers per se, as it is recognized that they can and do play an important role in primary health care. The question of the extent to which traditional healers, diviners and religious leaders can play a positive role in helping to prevent the harms generated by witchcraft beliefs is an important one requiring further research. While this paper focuses on Papua New Guinea, sorcery beliefs are widespread in many places worldwide. (*3*) Even in high-income countries, there has been increased recognition of the harms generated by witchcraft beliefs. For example, in 2012, United Kingdom of Great Britain and Northern Ireland published a national action plan to tackle child abuse linked to faith or belief, which includes concepts of witchcraft and spirit possession. (*4*) However, at present, sorcery beliefs in the provision of health services receive hardly any attention or resources.

## CONTEXT

### Sorcery beliefs and violence in Papua New Guinea

In Papua New Guinea, the term “sorcery” is used to represent the belief that one human being is capable of harming another by magical or supernatural means, as well as the practices associated with that belief. (*2*) While not new, these beliefs have gained greater attention recently due to the harm that can follow accusations of sorcery and concerns that violence is spreading across the country. (*5*) The scope of harm caused by sorcery accusation-related violence (SARV) is unknown. Even when cases are reported, the fragmented nature of data collation in Papua New Guinea makes it difficult to obtain a reliable account of overall case numbers. A recent 4-year study on SARV that took place in four provinces of Papua New Guinea recorded 1039 cases affecting 1554 people. (*6*) Victims of SARV include men, women, young and elderly people across the socioeconomic spectrum. However, in some places, women are targeted and often are most severely impacted. (*1*, *6*)

The traumatic impacts of SARV are wide-ranging for accused individuals, their families and the affected community. The impacts on children are extremely severe, including: witnessing parents, close relatives or community members being tortured and killed; stigmatization in school and the community; and exclusion from school resulting in poor educational outcomes. (*5*) Such experiences can lead to feeling ashamed and isolated and living in fear. Also, children are often accused due to the belief that sorcery is passed down through bloodlines. Many children develop the belief that sorcery is real and accept torture (of accused persons) as the norm. (*7*)

Harm from SARV is compounded by the fact that it is not properly addressed by the legal system. (*8*) Despite adequate criminal legislation, cases often go unreported as witnesses or victims fear community retaliation, while others may not believe the police will take their complaints seriously. This lack of accountability and justice can contribute to a culture of impunity, where individuals feel empowered to continue perpetrating acts of violence. This promotes a cycle of fear and mistrust, potentially further spreading the risk of accusation and violence. (*9*)

### Sorcery beliefs and health in Papua New Guinea

Sorcery beliefs and accusations in Papua New Guinea tightly intertwine with health, as people often blame sickness and death on sorcery. (*10*) This has two main implications for public health professionals. First, it can significantly impact the ability of health-care workers and field epidemiologists to provide appropriate interventions. Sorcery beliefs may delay or deter people from seeking medical assistance or make them less likely to follow medical advice. (*11*) For example, if a community believes a child's death was caused by sorcery, people may be reluctant to allow a field epidemiologist to investigate its real cause, and instead call a “diviner” to ascertain if sorcery caused it. (*12*) This can make it difficult to identify the source of an infection or disease and delay the steps needed to prevent its further spread.

Second, sorcery beliefs can lead to violence following an incidence of sickness or death, where people blame the death or sickness on a particular individual. Individuals accused of sorcery may be attacked, killed or tortured. Accusations can spiral out of control, sometimes involving multiple deaths, property destruction and the dislocation of entire communities. (*13*) Also, health-care workers can inadvertently trigger accusations in their communications about the cause of death. One way this occurs in Papua New Guinea is through the common practice of telling people that illnesses are *sik bilong ples* (the sickness of the area), which is interpreted as culture-related and caused by sorcery. Health-care professionals may make such diagnoses due to a lack of biomedical explanations and appropriate tests, or to protect patient confidentiality if the patient died of an illness involving stigma, such as HIV and AIDS.

## ACTION

### Health-care workers and navigating sorcery beliefs in Papua New Guinea

The difficulties and challenges of sorcery beliefs facing health-care workers were the focus of the first-ever training workshop on this topic for field epidemiologists in Papua New Guinea, held in 2022. The workshop was part of the Field Epidemiology Training Program of Papua New Guinea (FETPNG). Programme fellows are health extension officers drawn from across Papua New Guinea. The workshop participants included 17 advanced field epidemiology fellows and three senior coordination staff who attended a voluntary 90-minute session facilitated by an expert in SARV in Papua New Guinea from the National Research Institute. The objectives of the workshop were to raise awareness among the fellows of the problem of SARV in Papua New Guinea and its link to health, introduce some helpful strategies to mitigate SARV risk in health care, and encourage discussion among the participants on the effective strategies they have developed, thus generating peer-to-peer learning.

The workshop was organized in response to consistent feedback from the fellows on their experiences with tension and occasional conflict in the community associated with sorcery beliefs. The fellows observed that these tensions often delayed or created barriers to outbreak response and public health intervention efforts. The workshop involved a presentation and a free discussion between the fellows focusing on their experiences of the impact of sorcery beliefs on their work, the challenges it presents, and their strategies for effectively addressing them. With their permission, the discussion was recorded, and relevant parts were transcribed.

## LESSONS LEARNED

During the workshop, five fellows confirmed the negative impacts of sorcery beliefs on their ability to work effectively. One made the following observations:

I grew up in the traditional way. There were always stories about sorcery… My dad said this isn’t true, so I started not to believe it. The mindset changed over time though in the village. The common things – tuberculosis, cancers – were being blamed on sorcery… but things changed over time. I finished school and went away. When I came back – sorcery was widespread again. I would advocate that there was no such thing. One patient came in vomiting blood – a health worker was talking sorcery… these are the people we rely on to change the way people are thinking. How do we impart knowledge to the community?

Another said:

There was one facility in a very remote place that was closed for 2 months. A health worker was diagnosed with something at the hospital and died, and the health centre closed down. The brothers-in-law killed four people who were suspected to be sorcerers. The facility was closed. What we did was discuss with the community how best to reopen the health centre. We had to compensate those who died. We settled this, and then the facility opened. We see the issues as health workers – we hear people coming in saying sorcery. What do we do? Maybe we can prevent the violence at the facility level.

Fellows explained that where sorcery was believed to have caused sickness or death in a family, or the wider community, it was hard for them to enter communities, convey risk factors and implement prevention and control measures.

During the session, the fellows provided recommendations for support needed to better address sorcery beliefs and related violence in Papua New Guinea’s health-care sector. These included:

educating health-care workers about how to navigate sorcery beliefs related to death, sickness and other health matters; for example, one fellow noted, “What would help is education at the health centre level – officers need to be trained; they should have an action plan – a *step by step* guide [when faced with sorcery accusations]”;
encouraging use of confirmatory tests to help dispel myths and confirm a medical cause/viewpoint; another fellow said, “Confirmatory tests could help dispel myths. One defaulter in the TB program – he died. Uncle came and said that I had caused this. But because I can prove why he died – that stopped the process”;
encouraging timely reporting of test results and death certificates; promptness dispels accusations and violence; for instance, a third fellow said, “One factor is that death certificate is delayed – so it’s not there in time to avert violence”; and
developing effective ways to communicate a diagnosis of sickness or communicate the causes of death to a family member in ways that avoid triggering sorcery accusations; for example, another fellow said, “Health comes into this – a sign of disease triggers an accusation. We can spend more time educating the community like what causes fever.”

## Discussion

The close connections between sorcery beliefs and health mean that equipping health-care workers and field epidemiologists with strategies to address these beliefs effectively is critical to delivering better health care, facilitating timely responses to public health events and helping to prevent violence related to sorcery accusations. The effects of beliefs in sorcery and witchcraft on health-service delivery should be examined in all countries where it is common for health, illness, disability and death to be explained by reference to such beliefs. There is a great need for properly funded pilots to trial interventions such as those recommended in the workshop in Papua New Guinea to assess those that are most effective, and to share such findings both nationally and internationally. This is an intended direction of research for the authors, and further publications are planned.
